# Supplementary material for: Static magnetic field stimulation of the supplementary motor area modulates resting-state activity and motor behavior
Source: Commun Biol. 2019 Oct 31;2:397. doi: 10.1038/s42003-019-0643-8 (PMC6823375; doi:10.1038/s42003-019-0643-8)
Supplement: Supplementary file 1 — Reporting Summary [file 42003_2019_643_MOESM1_ESM.pdf]

nature research

Corresponding author(s): Guglielmo Foffani

Last updated by author(s): Jul 23, 2019

Reporting Summary

Nature Research wishes to improve the reproducibility of the work that we publish. This form provides structure for consistency and transparency in reporting. For further information on Nature Research policies, see Authors & Referees and the Editorial Policy Checklist.

Statistics

For all statistical analyses, confirm that the following items are present in the figure legend, table legend, main text, or Methods section.

n/a	Confirmed	
☐	☒	The exact sample size (*n*) for each experimental group/condition, given as a discrete number and unit of measurement
☐	☒	A statement on whether measurements were taken from distinct samples or whether the same sample was measured repeatedly
☐	☒	The statistical test(s) used AND whether they are one- or two-sided<br>*Only common tests should be described solely by name; describe more complex techniques in the Methods section.*
☐	☒	A description of all covariates tested
☐	☒	A description of any assumptions or corrections, such as tests of normality and adjustment for multiple comparisons
☐	☒	A full description of the statistical parameters including central tendency (e.g. means) or other basic estimates (e.g. regression coefficient) AND variation (e.g. standard deviation) or associated estimates of uncertainty (e.g. confidence intervals)
☐	☒	For null hypothesis testing, the test statistic (e.g. *F*, *t*, *r*) with confidence intervals, effect sizes, degrees of freedom and *P* value noted<br>*Give P values as exact values whenever suitable.*
☒	☐	For Bayesian analysis, information on the choice of priors and Markov chain Monte Carlo settings
☒	☐	For hierarchical and complex designs, identification of the appropriate level for tests and full reporting of outcomes
☒	☐	Estimates of effect sizes (e.g. Cohen's *d*, Pearson's *r*), indicating how they were calculated

Our web collection on statistics for biologists contains articles on many of the points above.

Software and code

Policy information about availability of computer code

Data collection	ePrime (behavioural data collection)
Data analysis	Matlab, Statistica

For manuscripts utilizing custom algorithms or software that are central to the research but not yet described in published literature, software must be made available to editors/reviewers. We strongly encourage code deposition in a community repository (e.g. GitHub). See the Nature Research guidelines for submitting code & software for further information.

Data

Policy information about availability of data

All manuscripts must include a data availability statement. This statement should provide the following information, where applicable:

- Accession codes, unique identifiers, or web links for publicly available datasets
- A list of figures that have associated raw data
- A description of any restrictions on data availability

Data for Fig. 2 and Fig. 3 are provided at https://osf.io/n3au4/

Field-specific reporting

Please select the one below that is the best fit for your research. If you are not sure, read the appropriate sections before making your selection.

☒ Life sciences ☐ Behavioural & social sciences ☐ Ecological, evolutionary & environmental sciences

Life sciences study design

All studies must disclose on these points even when the disclosure is negative.

Sample size	Sample sizes were conservatively determined based on common practice in the relevant literature
Data exclusions	Experiment 1a: One subject that received sham tSMS did not complete the "fully-cued" task and another subject that received sham tSMS did not complete the "uncued-compatible" task<br>Experiment 2: 1 subject was discarded due to excessive motion (>35% noisy samples) in at least one acquisition, so the final analyses were performed in 19 subjects
Replication	Experiment 1a was replicated in a within-subjects design with Experiment 1b.
Randomization	Allocation was randomized in all experiments
Blinding	All experiments were double-blind (except experiment 1c, which was single-blind)

Reporting for specific materials, systems and methods

We require information from authors about some types of materials, experimental systems and methods used in many studies. Here, indicate whether each material, system or method listed is relevant to your study. If you are not sure if a list item applies to your research, read the appropriate section before selecting a response.

Materials & experimental systems

n/a	Involved in the study	
☒	☐	Antibodies
☒	☐	Eukaryotic cell lines
☒	☐	Palaeontology
☒	☐	Animals and other organisms
☐	☒	Human research participants
☒	☐	Clinical data

Methods

n/a	Involved in the study	
☒	☐	ChIP-seq
☒	☐	Flow cytometry
☐	☒	MRI-based neuroimaging

Human research participants

Policy information about studies involving human research participants

Population characteristics	Experiment 1a: 20 subjects performed the tasks after receiving real tSMS (31.9±9.3 years old, 14 females) and 22 after receiving sham tSMS (31.2±8.4, 14 females). Real and sham groups were matched for age (unpaired t-test: p=0.82) and gender (two proportion test: p=0.66).<br>Experiment 1b: 16 of the subjects that participated in Experiment 1 (8 real, 8 sham) also repeated the experiment at least one week later in a double-blind crossover design (33.1±8.1 years old, 11 females).<br>Experiment 1c: we performed an additional single-blind behavioral experiment in 17 subjects (29.4±7.4 years old, 12 females) who received tSMS over the right M1. The new data were compared against the sham group of Experiment 1a, with no differences in age (p=0.47) or gender (p=0.65).<br>Experiment 2: We tested the functional aftereffects of 30-min tSMS of the SMA in a randomized double-blind sham-controlled crossover experiment in 20 subjects (28.5±5.2 years old, 9 females).
Recruitment	Participants were recruited among employees at the institution, students and friends
Ethics oversight	Comité Ético de Investigación de HM Hospitales

Note that full information on the approval of the study protocol must also be provided in the manuscript.

Magnetic resonance imaging

Experimental design

Design type	Resting state
Design specifications	10 minutes resting state (i.e. 250 fMRI volumes)
Behavioral performance measures	No behavioral measures were recorded within the MR

Acquisition

Imaging type(s)	T1w and 2D-EPI BOLD weighted fMRI
Field strength	3T
Sequence & imaging parameters	1) 3D T1-weighted MP-RAGE image with parameters TR/TE/TI 2300/3.34/900 ms, flip angle 8º, and isotropic spatial resolution 1mm3 (FoV: 256mm, matrix: 256x256, slice thickness: 1mm); 2) resting-state fMRI using a single-shot gradient-echo EPI 2D pulse sequence, with acquisition parameters TR/TE 2400/30 ms, optimum flip angle using the Ernst equation (i.e. 79º), isotropic spatial resolution of 3mm3 (FoV: 192mm, Matrix: 64x64, Slice Thickness: 3mm), and acceleration factor through parallel imaging x2 (IPAT2) – the acquisition of this sequence lasted 10 min giving rise to 250 fMRI volumes; 3) fieldmap generated from two 2D gradient echo images, with acquisition parameters TR/TE1/TE2 455/4.92/7.38 ms, flip angle 60º, and same spatial resolution as the fMRI acquisition.
Area of acquisition	Whole brain

Diffusion MRI ☐ Used ☒ Not used

Preprocessing

Preprocessing software	AFNI, FSL-FMRIB, ANTS
Normalization	Affine transformation plus diffeomorphic symmetric normalization (SyN) using ANTs toolbox
Normalization template	MNI152
Noise and artifact removal	Rigid motion correction, EPI distortion correction using a subject specific fieldmap. Physiological confounders were the 6 motion regressors and the average time series of white matter and ventricles masks. The derivative signals were also included.
Volume censoring	Censoring was based on three measures: frame-wise displacement, standard deviation, and DVARS

Statistical modeling & inference

Model type and settings	Fisher r-Z transformation was applied to all the seed based functional connectivity maps. Statistical analysis at the second level consisted in a random effects univariate analysis.
Effect(s) tested	Main effect and time X stimulation interaction

Specify type of analysis: ☒ Whole brain ☐ ROI-based ☐ Both

Statistic type for inference (See Eklund et al. 2016)	Voxel-wise
Correction	Main effect was thresholded at the voxel level (p<0.05, FWE corrected for multiple comparisons).<br>Interaction was thresholded at the voxel level puncorr<0.001, with FWE correction at the cluster level (pcluster<0.05).

Models & analysis

n/a	Involved in the study
☐	☒ Functional and/or effective connectivity
☒	☐ Graph analysis
☒	☐ Multivariate modeling or predictive analysis

Functional and/or effective connectivity	Functional connectivity was estimated using Pearson correlations
